# RETRACTED: Aburjania et al. Synthetic Makaluvamine Analogs Decrease c-Kit Expression and Are Cytotoxic to Neuroendocrine Tumor Cells. *Molecules* 2020, *25*, 4940

**DOI:** 10.3390/molecules31162902

**Published:** 2026-08-20

**Authors:** Zviadi Aburjania, Jason D. Whitt, Samuel Jang, Dwayaja H. Nadkarni, Herbert Chen, J. Bart Rose, Sadanandan E. Velu, Renata Jaskula-Sztul

**Affiliations:** 1 Department of Surgery, University of Alabama at Birmingham, 1824 6th Avenue S., Birmingham, AL 35233, USAjbrose@uabmc.edu (J.B.R.); 2 Department of Chemistry, University of Alabama at Birmingham, 901 14th Street S., Birmingham, AL 35294, USA; 3 O’Neal Comprehensive Cancer Center, University of Alabama at Birmingham, 1720 2nd Avenue South, Birmingham, AL 35294, USA

The journal retracts the article “Synthetic Makaluvamine Analogs Decrease c-Kit Expression and Are Cytotoxic to Neuroendocrine Tumor Cells” [1] cited above.

Following the publication of the article, concerns were brought to the attention of the Editorial Office regarding the integrity of figures presented in this publication [1].

In accordance with the journal’s standard procedures, the Editorial Office and Editorial Board conducted an investigation that identified indications of inappropriate editing in Figure 7. Although the authors cooperated with the investigation, the explanations and supporting materials provided were not deemed sufficient by the Editorial Board to resolve the identified concerns. As a consequence, the Editorial Board has lost confidence in the reliability of the reported findings and has decided to retract this publication in accordance with MDPI’s retraction policy.

This retraction was approved by the Editor-in-Chief of the journal *Molecules*.

The authors have been informed of this retraction and have indicated their agree-ment with this decision.
